# Case of Disease of the Brain

**Published:** 1858-08

**Authors:** B. Woodward

**Affiliations:** Galesburg, Ill.


ARTICLE IV.

### CASE OF DISEASE OF THE BRAIN.

BY B. WOODWARD, M. D.

Messrs. Editors,—The opportunities for making post-mortem examinations are so rare here in the West, that I am induced to trouble you with a slight history of a case and its revelations.

On the 31st of May last, I was called to see Willie, aged seven years, son of Rev. J. Blanchard, D. D. Has been in delicate health for several months, and for the last two months has exhibited a remarkable degree of drowsiness. He had been confined to his bed eight days, and been treated with domestic remedies by his parents. Found him with clean, moist tongue, rather pallid; pulse 100, and feeble; mind at times wandering; parotid gland on right side enlarged, and tender on pressure; right eye rather prominent with contracted pupil, the sclerotic coat infiltrated with serum, and having a dull, clouded appearance; vessels of the eye not enlarged; breathing free and natural, except rather hurried; bowels in a healthy condition; urine healthy in quantity and appearance; head very hot, and from right nostril there was a continual discharge of bloody serum.

The symptoms, conjoined with appearance of the child, which was: fair skin, soft silky hair, contracted chest, large head and small limbs—pointed me at once to struma, with tubercular deposit on the brain. What else could I make of it?

℞ Syr. iodide of iron and glycerin, and quinine et. pulv. Doveri.

The parents, not believing it possible that the case could be scrofula, discontinued the use of the medicine after giving two doses of each. Found him the next day with pulse 128, skin hot and mind more wandering, though the most of the time he lay in a semi-comatose condition. Ordered,

℞ Tinct. verat. vir. gtt. ij. every two hours till pulse reduced.

In six hours the pulse was brought down to 98, but the parents being afraid of the medicine, it also was discontinued. The right eye continued to protrude, and before the death of the patient (which took place on the night of the 8th of June), it was so much protruded that the lids could not cover it. The coma became most profound, and he had no more lucid intervals, and total blindness of both eyes obtained for the last two days of life. Spasms of both hands and arms, but particularly of the right arm and hand, were very constant for the last forty-eight hours.

Permission was granted to make an examination of the head, and twelve hours after death, in the presence of Drs. Hard, Taylor and McCurdy, I made the post-mortem examination.

Appearance of the body natural, the protrusion of the eye not so great as during life.

On removal of the calvarium, the dura mater was found very much congested, and all its vessels much enlarged. It was covered with points of tubercles, from the size of the head of a pin to that of a large pea, which, on being pricked, gave out pus. Removing the dura mater, large quantities of serum flowed out, and the arachnoid and pia mater were seen to be highly injected, and under the arachnoid were several spots of hæmorrhage, one of them near the top of the head as large as a twenty-five cent piece. Both arachnoid and pia mater were thickly covered with tubercles, the most of them softened. Removing now the brain, the whole base of the skull was found covered with pus, which was also oozing from innumerable points on the under surface of the brain. The bed of the optic thalami was occupied by a large abscess which had burst, and the pus had made a passage for itself along the right optic nerve into the cavity of the orbit. At the base of the cerebellum, at the superior portion of the medulla oblongata, we found a spot as large as a nutmeg completely disorganized, and cutting as low down on the cord as possible, it was found to be bathed in pus, and the membranes of the cord injected. The arachnoid had a dull, clouded appearance. Between the dura mater and the cribriform plate, was a collection of bloody serum, probably ℥j., which seemed to have exuded from a diseased portion of the dura mater, and it had found its way through the cribriform plate into the right nostril, all the branches of the olfactory nerve being saturated with the fluid. The ventricles were all filled with serum.

Such extensive disorganization of the brain is seldom found, and it was a matter of surprise to the physicians who witnessed it (and they are all men perfectly conversant with morbid anatomy), that life should have continued so long.

We had a perfect solution of all the morbid phenomena. The protrusion of the eye was due to the pressure of the abscess on the optic thalami, and the infiltration of the pus through the optic foramen.

The spasms of the arms and hands were doubtless caused by the disorganization of the medulla oblongata; and the escape of bloody serum from the nostrils, by the collection over the ethmoid bone.

It was a matter of regret that we had not permission to examine other organs, as it would have been interesting to have traced the tubercular disease through the system. As it is, it was most satisfactory in establishing the diagnosis of the case; and from the prominence of Doctor Blanchard, as President of Knox College, we hope we shall find it more easy in future to get permission to make post-mortem examinations.

It is a fearful fact, that strumous diseases are greatly on the increase. Risking the danger of being called "a hobby rider," I am compelled to pronounce many cases of disease "scrofulous" which have not been, and are not so called by many other practitioners, and the result of treatment seems to verify the diagnosis. With your permission, Messrs. Editors, I will before long send you the notes of a very interesting case of the kind.

GALESBURG, ILL., June 16th, 1858.

